# Relationship between wooden breast severity in broiler chicken, antioxidant enzyme activity and markers of energy metabolism

**DOI:** 10.1016/j.psj.2024.103877

**Published:** 2024-05-17

**Authors:** Binbin Li, Niina Kalmu, Xinyue Dong, Yuemei Zhang, Eero Puolanne, Per Ertbjerg

**Affiliations:** ⁎ Department of Food and Nutrition, University of Helsinki, Helsinki 00014, Finland; † Key Laboratory of Geriatric Nutrition and Health, Beijing Technology and Business University, Ministry of Education, Beijing 100048, China

**Keywords:** wooden breast, antioxidant enzyme, oxidative stress, citrate synthase, glycolytic potential

## Abstract

This study aims to provide new insight on the association between the development of wooden breast myopathy and mitochondrial and glycolytic activity under oxidative stress. Myopathic muscle had higher oxidative stress together with altered glycolytic metabolism and tricarboxylic acid (**TCA**) cycle. This was evidenced by significantly elevated antioxidant enzyme activities (catalase, superoxide dismutase, and glutathione peroxidase), decreased citrate synthase activity and postmortem glycolytic potential with increasing wooden breast severity. In addition, affected muscles also exhibited higher initial and ultimate pH values as well as reduced total glucose and lactate contents. Citrate synthase activity was negatively correlated to antioxidant enzyme activities. Taken together, we propose that the development of the wooden breast lesion is a chronic process that may be related to the failure of muscle fibers to defend against the excessively generated oxidative products promoted by mitochondrial damage accompanied by impaired TCA cycle. Furthermore, there was a positive correlation between citrate synthase activity and glycolytic potential, which suggests that the wooden breast condition is linked to the overall altered energy metabolism of the muscle, including the oxidative phosphorylation and glycolytic pathways.

## INTRODUCTION

Wooden breast, a growth rate-related myopathy which mostly occurs in the *pectoralis major* muscle of chicken, can be characterized by a palpably hard area accompanied by a visible pale and ridge-like bulge appearance, and the muscle surface is often covered with clear viscous fluid (Sihvo et al., 2014). Compared to normal muscles, besides the above-mentioned appearance alterations, muscles with wooden breast myopathy also exhibit decreased protein content together with increased collagen and lipid contents, poor water-holding capacity, and harder texture (Soglia et al., 2016; Li et al., 2023). These compromised quality related characteristics may be closely linked to changes of muscle architecture, where myofibers are replaced by accumulating interstitial connective tissue. This in turn is likely linked to muscle cell necrosis and a high degree of protein denaturation (Sihvo et al., 2014; Li et al., 2023). Consumers tend to reject these abnormal muscles due to their altered meat quality and the potential impact on animal welfare related to chicken health, and therefore the emergence and prevalence of this myopathy causes substantial economic losses to the chicken industry (Petracci et al., 2019). Moreover, Malila (2023) suggested that wooden breast affected muscle exhibiting elevated level of lipid and protein oxidation may increase the health risk for consumers as they become more exposed to oxidated food.

Hence, better understanding of the etiology and the onset of the wooden breast myopathy could help to find efficient ways to mitigate the incidence or alleviate the symptoms. In recent years, hypoxia, oxidative stress, and their interaction have been widely suggested as triggers of the wooden breast condition (Mutryn et al., 2015; Abasht et al., 2016; Sihvo et al., 2018; Malila et al., 2019; Petracci et al., 2019). Accordingly, Sihvo et al. (2018) suggested that hypoxia may occur at the initiation of the wooden breast lesion and induce further progression towards a severe condition of the myopathy. They reported that reduced vessel density and blood supply to the myofibers took place before the occurrence of macroscopic lesions. The development of wooden breast is possibly linked to ultrastructural alterations of the sarcoplasmic reticulum and mitochondria. Petracci et al. (2019) emphasized that the hypoxia condition can be exacerbated by the excessive growth of the *pectoralis major* muscle. Because of insufficient oxygen availability and metabolic waste removal, reactive oxygen species (**ROS**) are more likely to accumulate and trigger the oxidative stress under the hypoxic condition (Thanatsang et al., 2020). In comparison to normal muscles, wooden breast muscles exhibit higher ROS production and increased lipid and protein oxidation, suggesting that affected muscle suffers from oxidative stress (Pan et al., 2021; Li et al., 2022). Additionally, muscle with wooden breast lesion shows upregulated expression of hypoxia-related genes and proteins in response to the condition of oxidative stress and hypoxia (Mutryn et al., 2015; Malila et al., 2019; Thanatsang et al., 2020; Carvalho et al., 2023). To adapt to hypoxia, the cellular energy metabolism shifts from mitochondrial oxidative phosphorylation to the oxygen-independent glycolytic pathway for ATP generation by increasing the glycolytic flux and inhibiting the initiation of the tricarboxylic acid (**TCA**) cycle in mitochondria (Kierans and Taylor, 2021). On one hand, this shift may result in lower post-mortem glycolytic potential and consequently cause higher ultimate pH value in wooden breast muscle (Alnahhas et al., 2023). On the other hand, this shift with impaired mitochondrial activity may cause more ROS accumulation, thereby increasing the oxidative stress in muscle (Malila et al., 2019). Moreover, Abasht et al. (2016) suggested that the changes in glycogen content and glycolysis of wooden breast muscle may arise from a perturbation of carbohydrate metabolism in response to elevated ROS exposure, which in turn can contribute to the oxidative stress and progression of wooden breast myopathy.

Furthermore, to protect muscle cells from the oxidative damage induced by overproduced ROS, muscle fibers also contain multiple layers of antioxidant defense systems, such as the enzymes superoxide dismutase (**SOD**), catalase (**CAT**) and glutathione peroxidase (**GPx)**. In chicken SOD mainly localizes in the cytosol and mitochondrial intermembrane space (**Cu,Zn-SOD**) and in the mitochondrial matrix (**Mn-SOD**), where it function to promote free radical detoxification by catalyzing the conversion of superoxide radicals into hydrogen peroxide and oxygen (Surai, 2016). Hydrogen peroxides and other lipid hydroperoxides can subsequently be decomposed to water by CAT (localized in the peroxisomes) and GPx (primarily localized in the cytoplasm and mitochondria) (Brieger et al., 2012; Surai et al., 2018). Pan et al. (2021) reported that the antioxidant enzymes in wooden breast muscle were activated to protect muscle fibers from oxidative damages and maintain the redox homeostasis. Taken together, we hypothesize that the changes in energy metabolism (glycolysis and mitochondrial oxidative phosphorylation) and antioxidant enzyme activity induced by oxidative stress are linked to the severity of wooden breast.

Although some studies have implicated that there are changes in the glycolytic pathway, mitochondrial function, and antioxidant enzyme activity in wooden breast muscle, the knowledge on the relationships between these changes and wooden breast development is limited. Therefore, this study focused on the relationships with increasing wooden breast severity and the changes of: 1) antioxidant enzyme activities indicated by CAT, SOD and GPx; 2) postmortem glycolytic potential; and 3) citrate synthase activity. The decrease in citrate synthase activity is an indicator of loss of intact mitochondria. In addition, the correlations between these parameters together with our previous results of lipid and protein oxidation were conducted to investigate the potential relationship to the development of wooden breast syndrome.

## MATERIALS AND METHODS

### Sample Preparation

In this study, a total of 56 male broilers (Ross 308) were terminated at the age of 41 d and were slaughtered in accordance with EC Regulation No 1099/2009, in the University of Helsinki Large Animal Research Unit facilities at Viikki Experimental Farm. After slaughtering and wooden breast severity evaluation, chicken *pectoralis major* muscle from 40 birds were categorized into 4 different wooden breast severity groups based on the level of hardness and the size of affected area of the breast muscle: normal, mild, moderate, and severe. Each category contained 10 animals. The growth conditions of the birds and the wooden breast evaluation method were described in detail in our previous studies (Li et al., 2022; Li et al., 2023).

The skin of the birds was cut open at the keel, followed by removal of the left breast muscle (*M. pectoralis major*) from the carcass for wooden breast evaluation and leaving the right breast muscle still attached. At 5 min postmortem, a sample for the initial pH value analysis was taken from the left breast muscle. Then, carcasses with the right-side breast muscle were packaged in plastic bags with feathers and skin on, cooled in ice water and then stored at 2 ± 1°C for post-rigor sampling. At 24 h postmortem, muscles for ultimate pH value and other analyses were obtained from the right-side breast muscle. Around 2 × 2 cm slices were cut in the central area of the breast muscle and immediately wrapped with aluminum foil and frozen in liquid nitrogen (for ultimate pH measurement, samples were taken from the same sampling place and size as from the left breast muscle). All the samples were stored at –80°C until analysis.

Due to the observations that the histological lesions (white striping, wooden breast, and spaghetti meat) gradually decrease moving from the skin-facing surface towards the inner section of the *pectoralis major* (Petracci et al., 2019), we took samples from the superficial (ventral) part and deep (dorsal) part. The activities of the antioxidant enzymes CAT, SOD and GPx were determined both in the superficial part and deep part of muscle samples. Other indexes such as initial and ultimate pH, citrate synthase activity, and total glucose and lactate content were investigated in the less damaged deep part, only.

### Initial and Ultimate pH

For the initial and ultimate pH measurement, 0.5 g muscle was homogenized with 5 mL of 5 mM Na-Iodoacetate and 150 mM KCl solution. Then, the pH determination was conducted in the homogenate using a pH meter (Seven GO Pro, Mettler-Toledo GmbH, Schwerzenbach, Switzerland) equipped with glass electrode (Mettler-Toledo Inlab 427, Mettler-Toledo GMBH, Schwerzenbach, Switzerland). Duplicates were made from each bird.

### Activities of Antioxidant Enzymes

Enzyme contained solution was extracted from both the superficial and deep part of breast muscle according to the method of Carvalho et al. (2017) with some modifications. In detail, 1 g muscle was homogenized with 7 mL precooled phosphate buffer (containing Na_2_HPO_4_ and KH_2_PO_4_, 50 mM, pH 7.0) by an IKA Ultra-Turrax T25 homogenizer (Labortechnik, Staufen, Germany) at 13,500 rpm for 30 s. After centrifugation at 10,000 × *g* at 4°C for 20 min, the supernatant was collected. The pellet was washed once by using 7 mL of the same precooled phosphate buffer, followed by homogenization at the same speed for 10 s and centrifugation, and the supernatant was collected and mixed with the previous one. Then, the mixed supernatant was filtered over glass wool, and the filtrate was used for the activity measurements of CAT, SOD, and GPx. The protein content of the filtrate was determined by RC DC Protein Assay Kit (Bio-Rad Laboratories, Hercules, CA) using BSA as standard. The activities of the enzymes were expressed as U per g of protein.

CAT activity was measured based on the method detailed described by Aebi (1974). Aliquots of 33 μL filtrate (kept in the ice-bath) were mixed with 967 μL of 11 mM H_2_O_2_. Absorbance at 240 nm and 20°C with a 3 min duration and 20 s interval was recorded immediately by a spectrophotometer (Shimadzu UV-1,800, Japan). One unit of CAT activity was defined as the amount of enzyme needed to decompose l μmol of H_2_O_2_ per min.

Activity of total SOD was measured based on the ability of SOD to inhibit the autoxidation of pyrogallol, which could be reflected by a delay formation of a yellow-colored product, as previously described by Marklund and Marklund (1974) and Mesa-Herrera et al. (2019). Briefly, 100 μL of extract was mixed with 2.85 mL of Tris-cacodylic buffer (50 mM, pH 8.2) with 1 mM DTPA in a cuvette. Then, 50 μL of 50 mM pyrogallol was added into mixture to initiate the reaction. After gently mixing, the cuvette was monitored spectrophotometrically at 420 nm at 25°C every 30 s for 6 min. The sample replaced by 100 μL of phosphate buffer was taken as control. One unit of SOD was defined as the amount of extract needed to inhibit the pyrogallol autoxidation by 50%.

GPx activity was measured by an assay kit (Catalog Number CGP1, Sigma-Aldrich, St. Louis, MO). An aliquot of 50 μL enzyme solution extract was added into a mixture containing 0.89 mL GPx assay buffer (50 mM Tris-HCl with 0.5 mM EDTA, pH 8.0) and 50 μL NADPH assay reagent (5 mM NADPH, 42 mM reduced glutathione and 10 units/mL of glutathione reductase). After mixing, the reaction was activated by addition of 10 μL 30 mM tert-Butyl Hydroperoxide solution. The reaction system without the sample (replaced by 50 μL of phosphate buffer) was considered as the blank. The decrease in NADPH absorbance of both sample and blank were recorded at 340 nm, 25°C for 1 min using a kinetic program of the spectrophotometer with 15 s of initial delay and 10 intervals. One unit of GPx was defined as the amount of extract needed to cause the formation of 1 μmol of NADP^+^ from NADPH per minute at pH 8.0 at 25°C in this reaction system. The calculation of GPx activity was based on the difference of absorbance between sample and blank and the extinction coefficient of NADPH at 340 nm (6.22 /mM/cm^−1^).

### Citrate Synthase Activity

Citrate synthase activity was determined by using a commercial kit (Catalog Number CS0720, Sigma-Aldrich) by monitoring the color intensity at 405 nm, which is generated from the reaction of citrate synthesis with DTNB present. The procedure and calculation methods were described in detail by Li et al. (2022).

### Contents of Total Glucose and Lactate

Contents of total glucose and lactate were measured from homogenate prepared by homogenizing 0.5 g still frozen muscle sample with 10 mL 1 M HCl by an IKA Ultra-Turrax T25 homogenizer at 13,500 rpm for 2 × 20 s. Then, 2 mL of homogenate was collected for total glucose and lactate analysis, respectively.

For total glucose content measurement, the collected homogenate was hydrolyzed in a heating cabinet (Memmert 700, Memmert GMBH, Schwabach, Germany) at 100°C for 2 h and cooled down to room temperature. After adjusting the pH to between 6.5 and 7.8 by using 2 M NaOH. One mL of the solution was centrifuged at 4,000 *g* for 5 min, and the supernatant was collected. Total glucose content was determined enzymatically using Roche diagnostic kit (no. 20767131322, Roche Diagnostics GMBH, Mannheim, Germany) and glucose as standard curve for calculation.

The homogenate collected for lactate analysis, without the hydrolysis, was adjusted to pH value of 10 directly with 2 M KOH. The procedures afterwards were same as total glucose determination, and the lactate content was determined following the instruction of Boehringer Mannheim diagnostic kit (no. 10139084035, R-Biopharm AG, Darmstadt, Germany) and calculated by using lactic acid as standard curve.

### Glycolytic Potential

Glycolytic potential was calculated according to Monin and Sellier (1985), based on the sum of glycogen, glucose-6-phosphate, glucose, and lactate using the following formula:

Glycolyticpotential=2(glycogen+glucose+glucose−6−phosphate)+lactate

According to Immonen and Puolanne (2000), the method determined the total glucose content of the hydrolyzed muscle sample, mainly glycogen, glucose-1-phosphate, glucose-6-phosphate, and free glucose. Therefore, the contents of glycogen, glucose-6-phosphate, and free glucose in the formula were considered as the sum of total glucose content of the sample in this study, and this we here called total carbohydrate, when applicable.

### Statistical Analysis

All data were analyzed using the IBM SPSS Statistics 26. Within each parameter, duplicates were run for each muscle. ANOVA using general linear model combined with Duncan test were conducted to evaluate the effect of wooden breast severity on each index and the significant difference of means at a level of *P* < 0.05. In addition, Two-way ANOVA was also done to analyze the interaction effects of wooden breast severity and sampling location on the activities of antioxidant enzymes. Pearson correlations (n = 10 of each wooden breast severity) were performed to find the associations between parameters of antioxidant enzyme activities, lipid and protein oxidation and citrate synthase activity (data from Li et al. (2022) from the same set of samples) as well as glycolytic potential.

## RESULTS

### Activities of Antioxidant Enzymes

**Figure 1:**
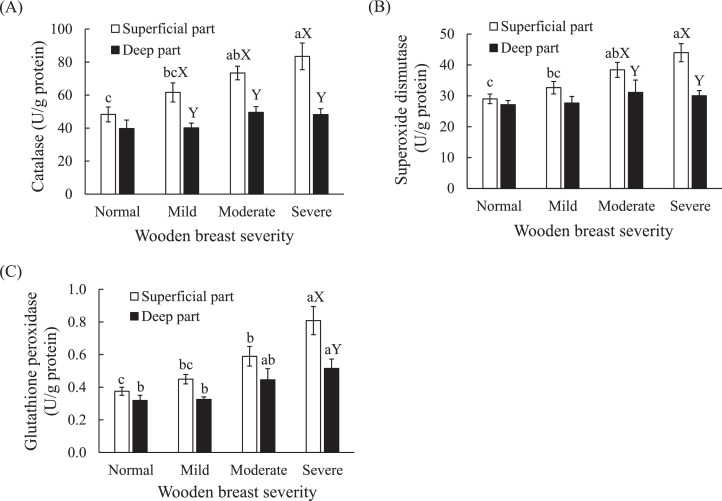
Effects of wooden breast severity on activities of antioxidant enzyme (catalase (A), superoxide dismutase (B) and glutathione peroxidase (C) in the superficial and deep part of *pectoralis major* muscle. One unit (U) of catalase activity was defined as the amount of extract needed to decompose l μmol of H_2_O_2_ per min. One unit of superoxide dismutase was defined as the amount of extract needed to inhibit the pyrogallol autoxidation by 50%. Glutathione peroxidase activity was expressed as the amount of extract needed to oxidize 1 μmol of NADPH per min at 25°C. Means ± standard errors are shown (n = 10). ^a–c^ Within location (superficial or deep), mean values with a common lowercase letter do not differ (*P* > 0.05). ^XY^ Within the same severity group, mean values marked with different capital letters differ significantly (*P* < 0.05).

Antioxidant enzymes are often used as markers of oxidative stress. Both wooden breast severity and sampling location had effects (*P* < 0.01) on the activities of antioxidant enzymes, whereas no interaction effects were observed (Table 1). Figure 1 presents how the activities of the antioxidant enzyme CAT, SOD, and GPx in the superficial and deep part of muscle were affected by the wooden breast severity. In the normal tissue, enzyme activities in the superficial part did not differ from those in the deep part (Figure 1, *P* > 0.05). As wooden breast severity increased, severely affected breast muscles exhibited the highest activities of CAT, SOD and GPx, reaching 83, 44, and 0.8 U, respectively. The activities increased remarkably in the superficial part compared to deep part. However, there was a higher activity of GPx in the deep part of severely affected muscle, only, compared to that of normal muscle (Figure 1C, *P* < 0.05), while no differences related to the activities of CAT and SOD were found in the deep part (Figure 1A and B, *P* > 0.05).

**Table 1:** Main effects of wooden breast severity, sampling location and their interaction on antioxidant enzyme activities, citrate synthase activity, initial and ultimate pH, total glucose and lactate content and glycolytic potential

Effects	WB severity	Location	WB severity * Location
Citrate synthase	**	/	/
Catalase	**	**	ns
Superoxide dismutase	**	**	ns
Glutathione peroxidase	**	**	ns
Initial pH	*	/	/
Ultimate pH	*	/	/
Total glucose	**	/	/
Lactate	*	/	/
Glycolytic potential	*	/	/

/: not applicable, ns: not significant, and WB: wooden breast.

*P < 0.05, **P < 0.01.

### Initial and Ultimate pH

**Figure 2:**
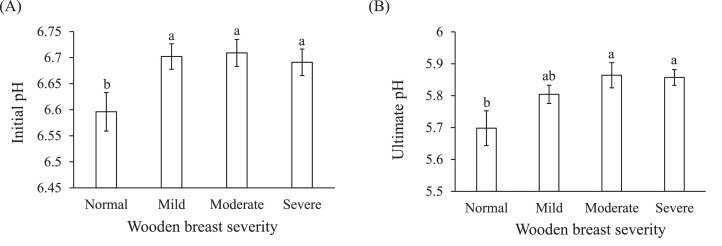
Changes of initial (A) and ultimate pH (B) in the *pectoralis major* muscle with different wooden breast severity. Means ± standard errors are shown (n = 10). a–c: within each indicator, mean values with the same letter do not differ (*P* > 0.05).

In this study, wooden breast severity showed significant main effects on initial and ultimate pH values of the muscles (Table 1). Moderate and severe wooden breast muscles had approximately 0.1 higher values of initial pH compared to normal muscle, whereas the ultimate pH was about 0.2 higher (Figure 2, *P* < 0.05).

### Total Glucose and Lactate Contents and Glycolytic Potential

**Figure 3:**
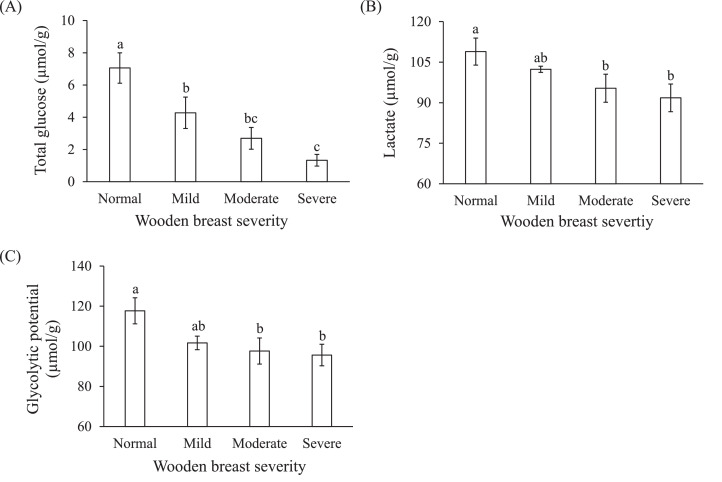
Effects of wooden breast severity on contents of total glucose (A), lactate (B) and glycolytic potential (C) in the *pectoralis major* muscle. Means ± standard errors are shown (n = 10). a–c: within each indicator, mean values with the same letter do not differ (*P* > 0.05).

The total glucose and lactate contents with increasing wooden breast severity are shown in Figure 3. Affected muscle showed lower level of total glucose (Figure 3A, *P* < 0.05), decreasing from 7.1 µmol/g in unaffected muscle to 1.3 µmol/g in severely affected muscle. A similar trend was also observed on lactate content, decreasing from 109 µmol/g in unaffected muscle to 92 µmol/g in muscle with severe lesion (Figure 3B, *P* < 0.05).

The glycolytic potential was calculated based on the sum of total glucose and lactate contents and was used as a marker to reflect the level of glycogen in the muscle. Wooden breast severity showed significant main effect on the level of glycolytic potential, which decreased by 19% (*P* < 0.05) from muscles without lesion (118 µmol/g) to severely affected (96 µmol/g) (Table 1 and Figure 3C).

### Citrate Synthase Activity

**Figure 4:**
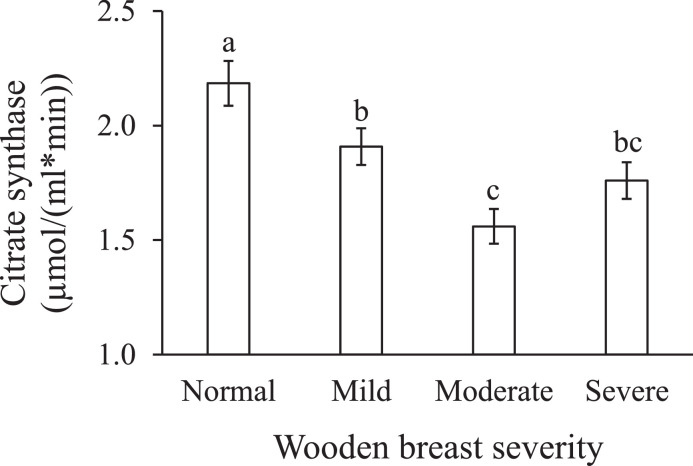
Effects of wooden breast severity on the activity of citrate synthase in the *pectoralis major* muscle. Means ± standard errors are shown (n = 10). a–c: mean values with the same letter do not differ (*P* > 0.05).

Citrate synthase is commonly used as a quantitative enzyme marker of intact mitochondria in skeletal muscle (Eigentler et al., 2012). In the present study, wooden breast severity affected the activity of citrate synthase in breast muscle (Table 1, *P* < 0.01). With the development of wooden breast, citrate synthase activity in nonwooden breast muscle was 2.2 µmol/(mL*min), and thereafter decreased down to 1.9, 1.6, and 1.8 µmol/(ml*min) towards increasing severity of wooden breast abnormality (Figure 4).

### Correlation Analysis

Besides the parameters we determined in this study, we also took the results of lipid and protein oxidation (indicated by TBARS, carbonyls and free thiols) from our previous study for correlation analysis. This may help us better understand the underlying reason for the changes, and the associations between oxidative stress with the progression of the wooden breast myopathy. As shown in Table 2, antioxidant enzyme activities indicated by CAT, SOD and GPx correlated positively to lipid and protein oxidation markers, where the coefficients to the TBARS value was 0.50, 0.55, and 0.47, respectively (*P* < 0.01), and to the formation of carbonyl groups was 0.35, 0.41, and 0.34, respectively (*P* < 0.05), and to the free thiol groups was −0.61, −0.47, and −0.58, respectively (*P* < 0.05). Furthermore, citrate synthase activity showed negative correlations with antioxidant enzymes (*r* = −0.50, −0.43, and −0.36, respectively), and a negative correlation to TBARS (*r* = −0.59, *P* < 0.01) as well as a positive correlation with free thiols (*r* = 0.50, *P* < 0.01). Similar correlations were also observed between glycolytic potential and the markers of antioxidant enzyme and lipid and protein oxidation. Moreover, there was a relationship between citrate synthase activity and glycolytic potential (*r* = 0.45, *P* < 0.01).

**Table 2:** Pearson correlations (n = 10 per wooden breast severity category) between the activities of antioxidant enzyme (CAT, SOD, and GPx), indicators of lipid and protein oxidation (TBARS, carbonyls and free thiols), and citrate synthase activity as well as glycolytic potential in the superficial part of *pectoralis major* muscle.

	CAT	SOD	GPx	Carbonyls	Free thiols	TBARS	CS	Gly.pot.
CAT	1							
SOD	0.41^⁎⁎^	1						
GPx	0.69^⁎⁎^	0.55**	1					
Carbonyls	0.35*	0.41^⁎⁎^	0.34*	1				
Free thiols	−0.61^⁎⁎^	−0.47^⁎⁎^	−0.58^⁎⁎^	−0.25	1			
TBARS	0.50^⁎⁎^	0.55^⁎⁎^	0.47^⁎⁎^	0.34*	−0.57^⁎⁎^	1		
CS	−0.50^⁎⁎^	−0.43^⁎⁎^	−0.36*	−0.03	0.50^⁎⁎^	−0.59^⁎⁎^	1	
Gly.pot.	−0.46^⁎⁎^	−0.34*	−0.42^⁎⁎^	−0.20	0.47^⁎⁎^	−0.46^⁎⁎^	0.45^⁎⁎^	1

CAT: catalase, SOD: superoxide dismutase, GPx: glutathione peroxidase, and CS: citrate synthase, Gly.pot.: glycolytic potential. * *P* < 0.05, ** *P* < 0.01.

## DISCUSSION

### Potential Association of Oxidative Stress With Wooden Breast Development

**Figure 5:**
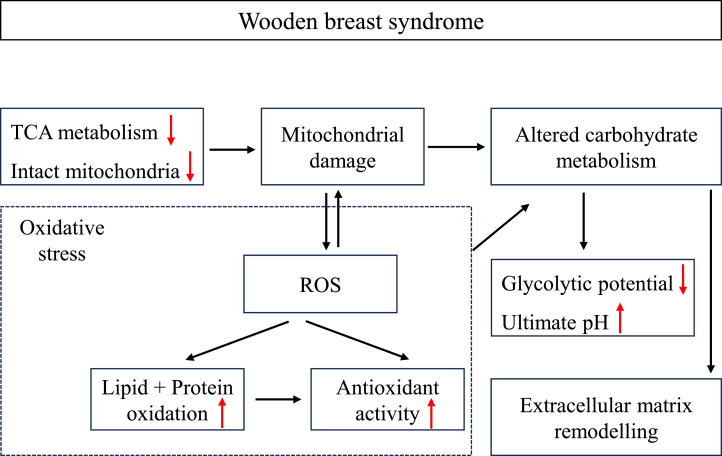
Hypothesis for the association between oxidative stress and the development of wooden breast syndrome, implicating altered energy metabolism. The accumulation of ROS in affected tissues is closely linked to mitochondrial damage, and oxidative stress occurs when activated antioxidant enzymes are insufficient to counteract the excessive free radicals and oxidative damage (dashed box). Furthermore, oxidative stress and mitochondrial damage may promote alterations in carbohydrate metabolism, thereby facilitating extracellular matrix remodeling in affected muscles. ROS: reactive oxygen species. TCA: tricarboxylic acid. ↑: increase. ↓: decrease.

Among the potential etiologic factors of wooden breast myopathy, oxidative stress has been widely considered as one factor closely related to the incidence and prevalence of this growth-rate related disease (Abasht et al., 2016; Petracci et al., 2019; Carvalho et al., 2023). In the current study, we propose a hypothesis on the association of oxidative stress with wooden breast development, implicating altered energy metabolism (Figure 5). Wooden breast affected chicken is likely to produce more ROS and free radicals while growing up (Praud et al., 2020). This over-production may be associated with the condition of hypoxia due to compromised blood and oxygen supply for the myofibers (Sihvo et al., 2018; Greene et al., 2019). Mitochondria, as the primary source of cellular ROS, showed damaged architecture with degenerated cristae and dissolved matrix in abnormal muscles (Papah et al., 2017), which may contribute to the generation of ROS (Xing et al., 2021). Citrate synthase is the first enzyme involved in the TCA cycle, and its activity is considered as one factor that can control the rate of the TCA cycle (Krebs, 1970). Additionally, the activity of citrate synthase in the present study was also served as a marker reflecting the level of intact mitochondria in lesioned muscles. Significantly decreased activity of citrate synthase was observed with increasing wooden breast severity (Figure 4), indicating that wooden breast muscle has lower activity of the mitochondrial TCA cycle and decreased level of intact mitochondria. A similar reduction in citrate synthase activity in wooden breast muscle was also reported by Shakeri et al. (2023). Our observation is in line with Papah et al. (2018), who reported that downregulation of the citrate synthase gene indicated decreased mitochondrial content and reduced TCA pathway in the muscle of affected chickens, suggesting a compromised bioenergetics capacity of the *pectoralis major* muscle. Furthermore, significant positive correlations between the loss of citrate synthase activity and increased level of indicators of lipid and protein oxidation and antioxidant enzymes (Table 2) may suggest that oxidative stress in the affected muscles is related to inhibited TCA and loss of intact mitochondria. Thomas and Ashcroft (2019) pointed out that in the context of inhibition of the TCA cycle, the subsequent electron transport chain may also be suppressed and subsequently prompting the formation of ROS. These produced ROS then have ability to rapidly damage the inner mitochondrial membrane and the oxidative phosphorylation, consequently resulting in mitochondrial dysfunction and ROS over-production (Park et al., 2011). Besides, mitochondria can also be attacked by outside ROS facilitating ROS release from mitochondria (Zorov et al., 2014). The released ROS could stimulate neighboring mitochondria to produce and release more ROS, eventually increasing the oxidative stress throughout the whole cell (Park et al., 2011). Moreover, Zhang et al. (2023) found that wooden breast muscle had significantly higher gene expression level of mitochondrial antioxidant factors in comparison to normal muscle, indicating an altered redox hemostasis of mitochondria in affected muscle. In addition, muscle fibers contain multiple layers of antioxidant defenses to prevent them from oxidative damage by scavenging ROS and free radicals. Increased activities of antioxidant enzymes with increasing wooden breast severity were observed in the current study, suggesting that the more severe the wooden breast condition is, the more oxidation it suffers. However, antioxidant defense systems are not always sufficient to maintain the balance of redox homeostasis (Brieger et al., 2012). Once muscle fibers lose control over oxidants production, oxidative stress will occur and thereby increase the likelihood of oxidative deterioration of its vulnerable components. Besides inducing oxidation of lipid and protein (Li et al., 2022), the activity of mitochondria and glycolysis may also be associated with the oxidative status of wooden breast muscle.

Moreover, the loss of intact mitochondria accompanied in wooden breast muscle not only can contribute to the oxidative stress but also may promote a shift in energy metabolism toward a more glycolytic pathway (Goda and Kanai, 2012; Alnahhas et al., 2023). However, the lower glycolytic potential (Figure 3) and higher ultimate pH (Figure 2) suggests an altered glycolytic metabolism in wooden breast muscle. Abasht et al. (2016) ascribed a lower glycogen and glycolytic potential level in affected muscle to an altered carbohydrate metabolism. In their study, they proposed that glucose utilization rerouted from glycolysis to pathways that support oxidative stress resistance and extracellular matrix remodeling.

Taken together, as illustrated in Figure 5, wooden breast syndrome is linked to imbalanced redox homeostasis and altered energy metabolism. The overall increase in antioxidant enzyme activities as well as lipid and protein oxidation indicate an accumulating oxidative environment in wooden breast muscle (dashed box). Under the oxidative stress condition, mitochondrial damage, as indicated by inhibited TCA and loss of intact mitochondria, can be accelerated due to an increased level of ROS, which may in turn exacerbate the oxidation level of muscles. Additionally, excessive oxidative stress together with mitochondrial damage may promote the perturbation of carbohydrate metabolism in affected muscle, causing a lower postmortem glycolytic potential and facilitating extracellular matrix remodeling, thereby contributing to the development of wooden breast syndrome.

### Effects of Wooden Breast Condition on the Activities of Antioxidant Enzymes

In the present study, the activities of antioxidant enzymes were used as a marker to reflect the oxidative status of chicken muscle. We observed a significant effect towards greater antioxidant enzyme activities with increasing wooden breast severity, and changes in the superficial part were remarkably more pronounced than in the deep part. These results imply that abnormal muscle, especially in the superficial part, was exposed to a high level of oxidation. The higher activities of CAT, SOD, and GPx suggests that the antioxidant defense system was activated to protect the muscles from oxidative damage as well as to maintain the redox homeostasis. Elevated activities of CAT, SOD, and GPx in wooden breast birds were also reported by Pan et al. (2021) and Xing et al. (2021). They suggested that antioxidant enzyme activities were enhanced in response to minimize the excessive production of ROS. Compared to normal birds, those with myopathic defect are more likely to generate and accumulate ROS during the growing (Praud et al., 2020), which will subsequently cause muscle oxidation. Our previous study reported that the higher the degree of wooden breast, the more intensive is the oxidative changes as evidenced by increasing lipid and protein oxidation (Li et al., 2022). The present results showed positive correlations between lipid and protein oxidation markers and activities of antioxidant enzymes (Table 2) with the development of wooden breast myopathy. Therefore, in comparison to normal or mildly affected muscle, moderately and severely affected exhibited greater activity of CAT, SOD, and GPx (Figure 1), which could be a response from the muscle fibers attempting to reduce increased oxidative damage caused by high exposure level to ROS and free radicals. In agreement, Carvalho et al. (2023) by applying proteomic analysis found that the endogenous antioxidant defense at the protein level was elevated in severely affected breast muscle and argued that this was to defend against oxidative stress and control the redox status. Furthermore, upregulated expression in wooden breast compared to normal muscles of antioxidant enzyme-related genes, such as SOD and GPx, has been reported by Thanatsang et al. (2020) and Pan et al. (2021). Collectively, we can conclude that there is evidence to support that increasing activity or expression of endogenous antioxidant enzymes is a reaction of the muscle to alleviate the oxidative damage continuously accumulating with wooden breast severity.

In wooden breast muscle, the progression of the lesion gradually became less from the surface towards the deep layer (Soglia et al., 2016). In agreement, Li et al. (2023) reported that the superficial part of wooden breast muscle exhibited pronounced reduction in total myofiber area accompanied with intensive cell necrosis, while less changes were observed in deep part. Accordingly, the overall difference of antioxidant enzyme activities between the superficial and deep part can be attributed to this uneven distribution of the wooden breast condition in breast muscle. The higher activities of antioxidant enzymes demonstrated that the superficial part of the muscle undergoes more oxidative damage, which is also supported by the results from our previous study that the superficial part has a higher level of lipid and protein oxidation than the deep part (Li et al., 2022).

### Correlations Between pH, Glycolytic Potential, and Citrate Synthase Activity

Wooden breast muscle presented higher values of both initial and ultimate pH. Similar results regarding ultimate pH were also observed by Baldi et al. (2019), Baldi et al. (2020) and Xing et al. (2020). As an important factor related to meat quality, information of pH value changes with wooden breast development may help us understand more on post-mortem quality changes such as effects on hardness (Ertbjerg and Puolanne, 2017). However, the typical hardness of wooden breast muscle is not due to sarcomere hypercontraction associated with higher ultimate pH (Baldi et al., 2020). Higher post-mortem pH value is likely due to disturbed glycolytic metabolism related to reduced glycogen level in wooden breast affected muscle (Mudalal et al., 2015; Abasht et al., 2016; Baldi et al., 2020). In agreement, the current study also found an altered glycolytic pathway reflected by decline of total glucose, lactate content and glycolytic potential with increasing wooden breast severity (Figure 3). Moreover, as expected, pH values in wooden breast muscle were negatively correlated to total glucose and lactate and glycolytic potential, respectively (Table 3). Reduced content of total glucose and glycolytic potential indicate that limited supply of substrates (glucose and glycogen) for glycolysis will subsequently result in an early cessation of this metabolism and less production of lactate postmortem (Baldi et al., 2020; Alnahhas et al., 2023). Higher ultimate pH value of wooden breast muscle thereby reflects a lower lactate accumulation. The higher initial pH (Figure 2A) measured 5 min post-mortem might be linked to the enhanced lactate export via monocarboxylate transporter 4 from the muscle fibers of live birds (Zhao et al., 2020). In the current study, the activity of citrate synthase decreased with increasing wooden breast severity, suggesting decreased TCA cycle activity of affected chicken. This may reflect an overall lower level of mitochondria in wooden breast muscle. Significantly negative correlations were observed between pH values and citrate synthase activity (*r* = −0.61 and −0.50, respectively, Table 3). Alnahhas et al. (2023) documented that mitochondrial damage in wooden breast myopathic muscle may potentially contribute to the higher ultimate pH.

**Table 3:** Pearson correlations between the initial and ultimate pH, citrate synthase activity, total glucose, and lactate content as well as glycolytic potential of *pectoralis major* muscle.

	Initial pH	Ultimate pH	Glucose	Lactate
Citrate synthase	−0.61^⁎⁎^	−0.50^⁎⁎^		
Glucose	−0.49^⁎⁎^	−0.60^⁎⁎^	1	
Lactate	−0.32*	−0.52^⁎⁎^	0.37*	1
Glycolytic potential	−0.46^⁎⁎^	−0.67^⁎⁎^	0.56^⁎⁎^	0.87^⁎⁎^

*P < 0.05, **P < 0.01.

In agreement with our data, Abasht et al. (2016) and Lake and Abasht (2020) reported that the utilization of glucose was changed in wooden breast *pectoralis major* muscle, and that the glucose was more likely used for extracellular matrix remodeling instead of glycogen synthesis, thereby accounting for the lower content of glycogen in lesion muscle. Alnahhas et al. (2023) concluded in their review that for an adaption to the hypoxic status, the energy metabolism in wooden breast birds shifts to a glycolytic-based pathway. This may lead to a premature depletion of glycolytic reserves, which could also partly explain the reduction in post-mortem glycolytic potential in abnormal breast.

In summary, the changes in pH values in wooden breast muscle may be affected by multiple factors linked to perturbed ante- and post-mortem metabolism pathways. The decreased glycolytic potential combined with lower lactate accumulation and decreased intact mitochondria contribute to the abnormal increase in pH value of wooden breast affected muscle.

## CONCLUSIONS

Activities of antioxidant enzymes of SOD, CAT, and GPx increased significantly with increasing wooden breast severity in the superficial part of muscle, whereas no pronounced changes occur in the deep part. These results indicate that wooden breast muscle, especially in the superficial part, suffers from a higher degree of oxidative stress compared to normal muscle. Wooden breast muscle shows greater initial and ultimate pH linked to a decrease of the glycolytic potential. Together with lower contents of post-mortem total glucose and lactate this suggests that wooden breast muscle have compromised glycolytic metabolism. Citrated synthase activity decreases with increasing wooden breast severity, indicating a loss of intact mitochondria in *pectoralis major* muscle accompanied by TCA cycle inhibition. Furthermore, antioxidant enzyme activities correlate negatively to citrate synthase activity and glycolytic potential. We thus conclude that the occurrence of oxidative stress in abnormal muscle is associated with a lower level of intact mitochondria coupled with inhibited TCA pathway. This may in turn promote the disturbance of carbohydrate metabolism causing wooden breast exacerbation and lower glycolytic potential.

## DISCLOSURES

The authors declare no conflicts of interest.
